# Safety of lecanemab in real-world patients with Alzheimer’s disease: a prospective, observational case series supported by FAERS signal analysis

**DOI:** 10.3389/fninf.2026.1977226

**Published:** 2026-10-02

**Authors:** Haixia Ji, Yang Yang, Xingqi Wu, Erhao Bao

**Affiliations:** 1 Department of Neurology, The First Affiliated Hospital of Anhui Medical University, Hefei, Anhui, China; 2 Department of Traditional Chinese Medicine, Sichuan Provincial People's Hospital East Sichuan Hospital & Dazhou First People's Hospital, Dazhou, Sichuan, China; 3 Department of Urology, Sichuan Provincial People’s Hospital East Sichuan & Dazhou First People’s Hospital, Dazhou City Traditional Chinese Medicine Industry Incubation Center, Dazhou City Traditional Chinese Medicine Research Center, Dazhou, Sichuan, China

**Keywords:** Alzheimer’s disease, FAERS, lecanemab, prospective study, signal analysis

## Abstract

**Objective:**

Lecanemab, a humanized immunoglobulin monoclonal antibody, has been approved for the treatment of Alzheimer’s disease (AD). Although clinical trials have demonstrated its favorable safety profile, real-world studies are crucial for identifying rare adverse events (AEs) and evaluating post-marketing safety. This study aimed to evaluate the safety of lecanemab by combining a clinical prospective, observational case cohort with data from the FAERS database, thereby providing a robust basis for the identification of potential safety signals.

**Methods:**

A prospective and observational study was conducted on patients with mild-to-moderate AD receiving lecanemab treatment at a tertiary medical center in China from July 2024 to February 2026. Extracted data included demographic characteristics, time to onset of AEs, and clinical symptoms. Exposure-adjusted incidence rates were calculated per 100 patient-years (PYs). Disproportionality analysis was performed using the US Food and Drug Administration Adverse Event Reporting System (FAERS), and safety signals for AEs observed in the cohort were evaluated utilizing reporting odds ratios (RORs) and proportional reporting ratios (PRRs). Additionally, data from these two real-world sources were compared and cross-validated.

**Results:**

The single-center prospective study enrolled a total of 91 patients. The median duration of lecanemab treatment was 185 days. Thirteen AEs were recorded (56.48/100 PYs), with the most frequent being amyloid-related imaging abnormalities-hemosiderin deposition (ARIA-H; defined as microhemorrhages and hemosiderin deposition) (12.39/100 PYs) and infusion-related reactions (IRRs) (8.85/100 PYs). In the FAERS database, the most frequently reported preferred terms (PTs) included headache, amyloid-related imaging abnormalities-edema/effusion (ARIA-E), ARIA-H, and IRRs. The majority of AEs occurred within the first 1–2 months after initiating lecanemab treatment (*n* = 393, 45.65%). Furthermore, FAERS analysis revealed a potential safety signal for cerebral infarction. However, this finding must be interpreted cautiously due to the lack of complete data on traditional stroke risk factors and concomitant antithrombotic use in spontaneous reports.

**Conclusion:**

In this single-center prospective observational study, lecanemab demonstrated a generally favorable safety profile. Patients with AD aged 65 to 85 years represent a high-risk demographic for AEs. Particular clinical vigilance is required regarding the occurrence of AEs within the first two months of lecanemab initiation. Notably, ARIA (including both ARIA-E and ARIA-H) can occur at any phase during the treatment course.

## 1 Introduction

Alzheimer’s disease (AD) is the most common type of dementia, accounting for approximately 60 to 80% of global dementia cases (Author, 2024). It is a progressive neurodegenerative disease initially presenting with memory decline, which gradually impairs thinking, language, and problem-solving abilities, ultimately leading to severe cognitive impairment, neuropsychiatric symptoms, and loss of social function (Author, 2024). The core pathological hallmarks of AD include the excessive extracellular deposition of amyloid-beta (Aβ) plaques, intracellular neurofibrillary tangles formed by hyperphosphorylated tau proteins, accompanied by neuronal loss, synaptic dysfunction, and neuroinflammatory responses. These pathological changes typically originate in the entorhinal cortex and hippocampus—regions critical for memory formation (Knopman et al., 2021; Scheltens et al., 2021). Currently, there are approximately 57 million patients with AD worldwide, a number projected to exceed 150 million by 2050, with over 60% of cases occurring in developing countries (GBD 2019 Dementia Forecasting Collaborators, 2019). The situation in China is particularly severe. According to the China Alzheimer’s Disease Report 2025, there are nearly 17 million available cases of AD and other forms of dementia in China, with a prevalence slightly higher than the global average that continues to rise with advancing age (Zhi et al., 2025). With the exacerbation of population aging, the incidence of AD is continuously increasing, imposing a heavy economic and societal burden on patients, families, and healthcare systems (Tay et al., 2024).

For a long time, the treatment of AD has predominantly relied on symptomatic medications, including acetylcholinesterase inhibitors (AChEIs) such as donepezil, rivastigmine, and galantamine, as well as the glutamate receptor antagonist memantine (Li et al., 2018; Karimi Tari et al., 2024). Donepezil improves cognitive function by inhibiting acetylcholinesterase activity, thereby increasing the concentration of acetylcholine in the synaptic cleft. Memantine, as a non-competitive N-methyl-D-aspartate (NMDA) receptor antagonist, blocks neurotoxicity caused by glutamatergic overexcitation (Li et al., 2018; Karimi Tari et al., 2024). However, the efficacy of these conventional drugs is significantly limited: they only provide temporary alleviation of cognitive decline and neuropsychiatric symptoms, fail to target the underlying pathogenic mechanisms, and are unable to halt disease progression (Kim et al., 2024). As the disease progresses to moderate-to-severe stages, the effectiveness of conventional medications gradually diminishes, and the cumulative effects of adverse drug reactions complicate the assessment of the benefits and risks of continued treatment (Glynn-Servedio and Ranola, 2017). Furthermore, real-world studies indicate that long-term adherence to conventional anti-dementia drugs is suboptimal; although the average persistence rate for memantine (61%) is higher than that for AChEIs (46%), the overall discontinuation rates remain substantial (Sistanizad et al., 2025; Cummings et al., 2016). Against this backdrop, novel disease-modifying therapies (DMTs) targeting the fundamental etiology of the disease have emerged (Hering et al., 2025).

Lecanemab is a humanized immunoglobulin G1 (IgG1) monoclonal antibody that directly targets and clears aggregated soluble and insoluble Aβ, thereby reducing cerebral amyloid plaque burden and delaying the neurodegenerative process (Park et al., 2024). The confirmatory Phase III clinical trial, Clarity AD (NCT03887455), enrolled 1,795 patients with early-stage AD (898 in the lecanemab group and 897 in the placebo group). The results demonstrated that after 18 months of treatment, the lecanemab group exhibited a 27% slower rate of decline in the Clinical Dementia Rating-Sum of Boxes (CDR-SB) score compared to the placebo group. Additionally, it met all key secondary endpoints, including a significant reduction in Aβ-PET amyloid plaque burden and improvements in cognitive and functional assessments (van Dyck et al., 2023). Based on these findings, lecanemab received accelerated approval from the US Food and Drug Administration (FDA) on January 6, 2023, for the treatment of early AD with mild cognitive impairment or mild dementia. Subsequently, on July 6, 2023, the FDA approved its supplemental Biologics License Application (sBLA), making lecanemab the first fully FDA-approved anti-Aβ monoclonal antibody (Jin and Noble, 2024). On January 9, 2024, the drug was approved for marketing by the National Medical Products Administration (NMPA) of China for the treatment of mild-to-moderate AD (Li L. L. et al., 2025).

Amyloid-related imaging abnormalities (ARIA) are the most notable adverse events associated with lecanemab therapy (van Dyck et al., 2023). ARIA encompasses two main subtypes: ARIA-E, characterized by parenchymal edema or sulcal effusion, and ARIA-H, which includes microhemorrhages, macrohemorrhages, and superficial siderosis. These abnormalities represent a key safety consideration in clinical practice (Li L. L. et al., 2025). The core Clarity AD study and its subsequent open-label extension (OLE) demonstrated that lecanemab is generally well-tolerated over long-term treatment (van Dyck et al., 2023). The 36-month OLE data further confirmed that no new safety signals emerged with continued lecanemab treatment, and ARIA rates remained low after the first 6 months, with no association between ARIA occurrence and accelerated long-term clinical progression (van Dyck et al., 2025). During the core study plus open-label extension (Core + OLE) period, the most common adverse events (AEs) with an incidence exceeding 10% in the lecanemab group were, in descending order, infusion-related reactions (24.5%), ARIA-H (16.0%), COVID-19 (14.7%), ARIA-E (13.6%), and headache (10.3%) (van Dyck et al., 2023). ARIA-E and ARIA-H typically presented as mild to moderate on neuroimaging. ARIA-E usually occurred within the first 3 to 6 months of treatment initiation (van Dyck et al., 2025) and was more prevalent among apolipoprotein E (APOE) ε4 carriers (16.8% in heterozygotes, 34.5% in homozygotes, and 6.5% in non-carriers) (van Dyck et al., 2025; Qi et al., 2026). Similarly, a clear APOE ε4 gene-dose effect has been demonstrated for ARIA risk: compared with non-carriers, the relative risk of ARIA was 1.45 for heterozygotes and 3.54 for homozygotes (Qi et al., 2026). ARIA-H also showed a higher incidence in APOE ε4 carriers than in non-carriers (approximately 19% vs. 10% in the Clarity AD core study) (van Dyck et al., 2025). There were no lecanemab-related deaths during the core study period; however, nine deaths occurred during the OLE phase, four of which were considered potentially related to the study treatment (van Dyck et al., 2025). Among the 24 deaths reported during the Core + OLE period, three were attributed to intracerebral hemorrhage (ICH)—one in the placebo group during the core study, and two in patients receiving lecanemab during the OLE phase (one of whom concurrently received tissue plasminogen activator, and the other was on anticoagulant therapy) (van Dyck et al., 2025). Although clinical trial results support a favorable benefit–risk profile for lecanemab, isolated cases of severe ARIA-E events have been reported (Yamazaki et al., 2025), underscoring the need for continuous post-marketing safety monitoring in real-world clinical practice.

It is well recognized that due to the stringent inclusion and exclusion criteria of clinical trials, significant disparities exist between clinical trial populations and real-world patients. Real-world AD patients are often older, possess more comorbidities, and undergo more complex polypharmacy regimens, which may lead to discrepancies between the real-world safety profile of the drug and clinical trial outcomes (Qi et al., 2026). Furthermore, certain rare AEs or those with a prolonged latency period are typically identified only after the drug is marketed and utilized by a large-scale population (Iwata et al., 2026). With the ever-growing number of AD patients globally, it is particularly vital to evaluate the safety profile of lecanemab based on real-world data (GBD 2019 Dementia Forecasting Collaborators, 2019).

The US Food and Drug Administration Adverse Event Reporting System (FAERS) is a spontaneous reporting system database for adverse drug events (ADEs) maintained by the FDA (Li Z. et al., 2025). In recent years, disproportionality analysis based on the FAERS database has been widely employed for the early detection and adjunctive identification of post-marketing drug safety signals (Li Z. et al., 2025). Therefore, this study aimed to perform signal mining and analysis of lecanemab-related ADE reports within the FAERS database. Furthermore, this data was integrated with a single-center prospective, observational study to comprehensively evaluate the AEs and safety profile of lecanemab in patients with AD, thereby providing supplementary reference and evidence for safe clinical medication practice.

## 2 Materials and methods

### 2.1 Study design and patients

A total of 91 patients with mild-to-moderate Alzheimer’s disease (AD) (aged ≥ 18 years) who received lecanemab treatment at the Department of Neurology, The First Affiliated Hospital of Anhui Medical University, from July 2024 to February 2026, were consecutively enrolled. We prospectively tracked and recorded adverse events (AEs) occurring during their lecanemab treatment. The inclusion criteria were: (1) clinical diagnosis of AD according to the NIA-AA criteria (van Dyck et al., 2025); (2) clinician’s decision to initiate lecanemab treatment; and (3) voluntary provision of written informed consent. The exclusion criteria included: more than 4 microhemorrhages (defined as ≤10 mm at the greatest diameter), any single macrohemorrhage (>10 mm at greatest diameter), any area of superficial siderosis, concurrent use of anticoagulant medications, apolipoprotein E (APOE) ε4 homozygosity, evidence of vasogenic edema, and simultaneous participation in other clinical trials. These ARIA-related exclusion thresholds were applied in accordance with the CLARITY-AD trial (van Dyck et al., 2023). APOE ε4 heterozygotes were not excluded and were retained in the cohort. Lecanemab was administered via intravenous infusion at a dose of 10 mg/kg once every two weeks. The study protocol was approved by the Institutional Review Board (Approval No.: IRB2023001).

### 2.2 Data collection and definitions

Prior to treatment initiation, baseline demographic data, APOE genotype, and baseline brain magnetic resonance imaging (MRI) scans (serving as a reference for ARIA evaluation) were collected. All MRI scans were performed on 3-T scanners, and each patient was consistently scanned on the same machine throughout the follow-up period. ARIA-E was monitored using 3D-FLAIR (slice thickness 1.0 mm), and ARIA-H was evaluated using susceptibility-weighted imaging (SWI; minimum echo time ≥20 ms). Before and after each infusion, uniformly trained researchers recorded, categorized, and graded all AEs according to the Common Terminology Criteria for Adverse Events (CTCAE) version 5.0, published by the National Cancer Institute (NCI).

Throughout the treatment cycle, patients or their caregivers were instructed to document any symptoms of discomfort. If patients experienced any new-onset or worsening neurological symptoms (e.g., visual disturbances, focal neurological deficits, confusion, or seizures) on non-infusion days, they were required to contact the research team immediately and arrange for an emergency visit. All patients underwent regular brain MRI scans in accordance with standard clinical protocols (e.g., prior to the 5th, 7th, and 14th infusions, or at any time when suspicious symptoms of ARIA were presented). MRI scans were independently reviewed by two board-certified neuroradiologists with >10 years of experience in neurodegenerative disease imaging; both had completed dedicated ARIA training modules according to the ASNR consensus guidelines, and final decisions were reached by consensus. ARIA was graded according to the American Society of Neuroradiology (ASNR) consensus grading framework (Cogswell et al., 2025), which is identical to the ARIA grading scale used in CLARITY-AD (van Dyck et al., 2023). This scale was purposefully designed to be simple and can readily be used in routine clinical practice; more research-oriented scales, were therefore not considered necessary.

### 2.3 FAERS signal mining

The FAERS is an openly accessible database containing information on AEs and medication error reports submitted to the FDA. In accordance with the raw data deduplication method recommended by the FDA, the PRIMARYID, CASEID, and FDA_DT fields from the DEMO table were selected and sorted in the order of CASEID, FDA_DT, and PRIMARYID. For reports with identical CASEID, the one with the latest FDA_DT was retained. Furthermore, for reports with identical CASEID and FDA_DT, the one with the largest PRIMARYID was kept. Following these principles, raw data deduplication was executed using SAS software.

Duplicate reports (the same case/report submitted by different sources) and multiple reports (follow-up reports on the same case providing additional or updated information) were removed, ensuring only the latest version of each case/report was extracted. Subsequently, only AE cases/reports where the drug’s role was designated as “Primary Suspect” (PS) were included; reports where the drug was designated as “Concomitant” or “Interacting” were excluded.

Adverse events were coded and classified into System Organ Classes (SOC) and Preferred Terms (PT) utilizing the Medical Dictionary for Regulatory Activities (MedDRA) version 27.1. Lecanemab was approved by the FDA in July 2023 for the treatment of patients with early-stage AD, encompassing mild cognitive impairment or mild dementia. Therefore, OpenVigil 2.1 was utilized to extract AE reports from the third quarter of 2023 to the fourth quarter of 2025, with the indication restricted to “Alzheimer’s disease” (i.e., extracting “Alzheimer’s disease” at the PT level) and lecanemab set as the primary suspect drug. All aforementioned analyses and extractions were performed in R software.

### 2.4 Statistical analysis

In the real-world prospective, observational case series, the exposure-adjusted incidence rates of all AEs and treatment-emergent AEs were calculated based on the number of patients experiencing the event per 100 patient-years (PYs) of exposure time.

Exposure-adjusted incidence rate (per 100 PYs) = (Number of events / Total exposure years) × 100.

Disproportionality analysis was conducted by calculating the Reporting Odds Ratio (ROR) and the Proportional Reporting Ratio (PRR). Larger ROR and PRR values indicate stronger signals, implying a greater statistical association between the target drug and the target AE. Both metrics were calculated based on the parameters of a 2 × 2 contingency table for signal detection (Table 1), comparing the observed count of the target drug-AE combination against all other drugs and events in the database. The specific formulas and predefined threshold criteria for these two indicators are detailed in Table 2. Importantly, these disproportionality measures from FAERS are used solely for qualitative signal screening and are not numerically compared with the exposure-adjusted incidence rates derived from our cohort.

**Table 1:** 2×2 table for the signal detection.

Database	Target AE	All other AE	Total
Target drug	a	b	a + b
All other drugs	c	d	c + d
Total	a + c	b + d	a + b + c + d

a, number of reports containing both the target drug and target AE; b, number of reports containing other AE of the target drug; c, number of reports containing the target AE of other drugs; d, number of reports containing other drugs and other AE.

**Table 2:** Formulas and threshold values of ROR and PRR.

Method	Formulas	Inclusion criteria for AE
ROR	$\mathrm{ROR}=\frac{\left(a/c\right)}{\left(b/d\right)}$	95% > 1
	$\mathrm{SE}(\ln\mathrm{ROR})=\sqrt{\frac{1}{a}+\frac{1}{b}+\frac{1}{c}+\frac{1}{d}}$	a ≥ 3
	$95\%\mathrm{CI}=e^{\ln(\mathrm{ROR})\pm 1.96\sqrt{\frac{1}{a}+\frac{1}{b}+\frac{1}{c}+\frac{1}{d}}}$	X^2^ > 4
PRR	$\mathrm{PRR}=\frac{a/(a+b)}{c/(c+d)}$	95%CI > 1
	$\mathrm{SE}(\ln\mathrm{PRR})=\sqrt{\frac{1}{a}+\frac{1}{a+b}+\frac{1}{c}+\frac{1}{c+d}}$	a ≥ 3
	$95\%\mathrm{CI}=e^{\ln(\mathrm{PRR})\pm 1.96\sqrt{\frac{1}{a}+\frac{1}{a+b}+\frac{1}{c}+\frac{1}{c+d}}}$	X^2^ > 4

### 2.5 Time-to-onset (TTO) analysis

Furthermore, we conducted a time-to-onset (TTO) analysis, defined as the time interval between the initiation of lecanemab treatment and the onset of adverse drug reactions. This analysis elucidates the temporal association between the occurrence of AEs and drug administration, providing a temporal reference for clinical decision-making. Following treatment initiation, the incidence of AEs typically depends on the drug’s pharmacological mechanism and tends to fluctuate over time. Conversely, adverse reactions unrelated to the drug treatment occur at a constant background rate.

In this study, we utilized the median, quartiles, and the Weibull shape parameter (WSP) to assess the TTO. Statistical analysis of TTO was performed using the Weibull distribution test, which determines the rate of change in the incidence hazard of AEs. The Weibull distribution is characterized by two parameters: the scale parameter (α) and the shape parameter (β). The TTO data of adverse reactions were compiled into a Microsoft Excel dataset and imported into R software. The corresponding R script for the Weibull distribution test was executed to calculate these parameters.

ROR values and PRR values indicate the strength of the signal, with higher values indicating a stronger correlation between the target drug and the target adverse event (AE). The signal generation rules are as follows:

(1) Lower limit of the 95% confidence interval (CI) > 1.
(2) Number of reports (a) ≥ 3.
(3) Chi-squared analysis > 4.

## 3 Results

### 3.1 Characteristics of lecanemab-related adverse event reports

A total of 2,969 reports identifying lecanemab as the primary suspect drug were retrieved from the FAERS database. Among these reports, adverse events (AEs) were more frequently reported in females (53.3%) than in males (37.1%), with patients aged 65 to 85 years constituting the largest demographic group (64.6%). The principal reporters of lecanemab-related AEs were physicians (43.0%), and the vast majority of reports originated from the United States (81.8%) and Japan (14.5%).

The single-center prospective observational study enrolled a total of 91 patients receiving lecanemab treatment. Within this cohort, 34 patients (37.4%) were male, and the 65 to 85 years age group represented the largest proportion (*n* = 51, 56.0%). The majority of the patients (*n* = 61, 67.0%) were enrolled during the year 2025. Among the 91 included patients, a total of 13 experienced AEs. The median duration of lecanemab exposure was 185 days. The demographic profiles of the AE reports from the FAERS database, along with the baseline characteristics of the real-world patient cohort, are detailed in Table 3. Of note, FAERS percentages represent reporting proportions in spontaneous reports, whereas cohort percentages reflect baseline patient characteristics. These descriptive data are shown separately for contextual purposes and are not directly compared numerically between the two datasets.

**Table 3:** Basic information of adverse event reports related to lecanemab and single center patients.

Variable		FAERS		Single center	
		Number	Percentage	Number	Percentage
Sex	Male	1,102	37.1%	5	38.5%
	Female	1,583	53.3%	8	61.5%
	Missing	284	9.6%	-	-
Age	>85 years	73	2.5%	-	-
	65 ~ 85 years	1918	64.6%	10	76.9%
	18 ~ 64.9 years	248	8.4%	3	23.1%
	Missing	730	24.6%	-	-
APOE genotype	ε2/ε3	-	-	2	2.2%
	ε2/ε4	-	-	3	3.3%
	ε3/ε3	3	23.1%	35	38.5%
	ε3/ε4	10	76.9%	51	56.0%
Reporter	Consumer	679	22.9%	-	-
	Health Professional	375	12.6%	-	-
	Pharmacist	61	2.1%	-	-
	Physician	1,277	43.0%	13	100%
	Missing	577	19.4%	-	-
Outcome	Death	119	4.0%	-	-
	Disability	6	0.2%	-	-
	Hospitalization	623	21.0%	-	-
	Life-Threatening	30	1.0%	-	-
	Other(see footnote)	351	11.8%	7	53.8%
	Required intervention	9	0.3%	6	46.2%
	missing	1831	61.7%	-	-
Get Data Year	2023	242	8.2%	-	-
	2024	1,246	42.0%	3	23.1%
	2025	1,481	49.9%	10	76.9%
Reporter Country (Top three)	US	2,430	81.8%	-	-
	JP	431	14.5%	-	-
	CN	35	1.2%	13	100%
Time to SOC onset	0–30 days	182	21.1%	-	-
	31–60 days	211	24.5%	3	23%
	61–90 days	91	10.6%	1	7.7%
	91–120 days	51	5.9%	1	7.7%
	121–150 days	31	3.6%	1	7.7%
	151–180 days	31	3.6%	-	-
	181–360 days	146	17.0%	1	7.7%
	>360 days	118	13.7%	-	-
	Missing	2,108	-	6	46.2%

*“Other” includes serious adverse events that do not fall under the other outcome categories (death, disability, hospitalization, life-threatening, or required intervention). These events encompass a wide spectrum of clinical presentations, including ARIA (both oedema/effusion and microhaemorrhages/haemosiderin deposition), cerebrovascular events (infarction, hemorrhage, stroke), neurological and psychiatric symptoms, cardiovascular events, infections, systemic symptoms, musculoskeletal injuries, and infusion-related reactions, among others.

### 3.2 Top 20 adverse events by signal frequency for lecanemab

Disproportionality analysis was performed to evaluate safety signals for adverse events (AEs) where lecanemab was identified as the primary suspect drug. For enhanced visualization, a forest plot was generated to present the reporting odds ratios (RORs) and 95% confidence intervals (CIs) of the top 20 most frequent positive AEs associated with lecanemab for the indication of Alzheimer’s disease (Figure 1). The top 20 AE signals primarily comprised headache, ARIA-E, ARIA-H, infusion-related reactions (IRRs), chills, fatigue, and pyrexia (Figure 1).

**Figure 1:**
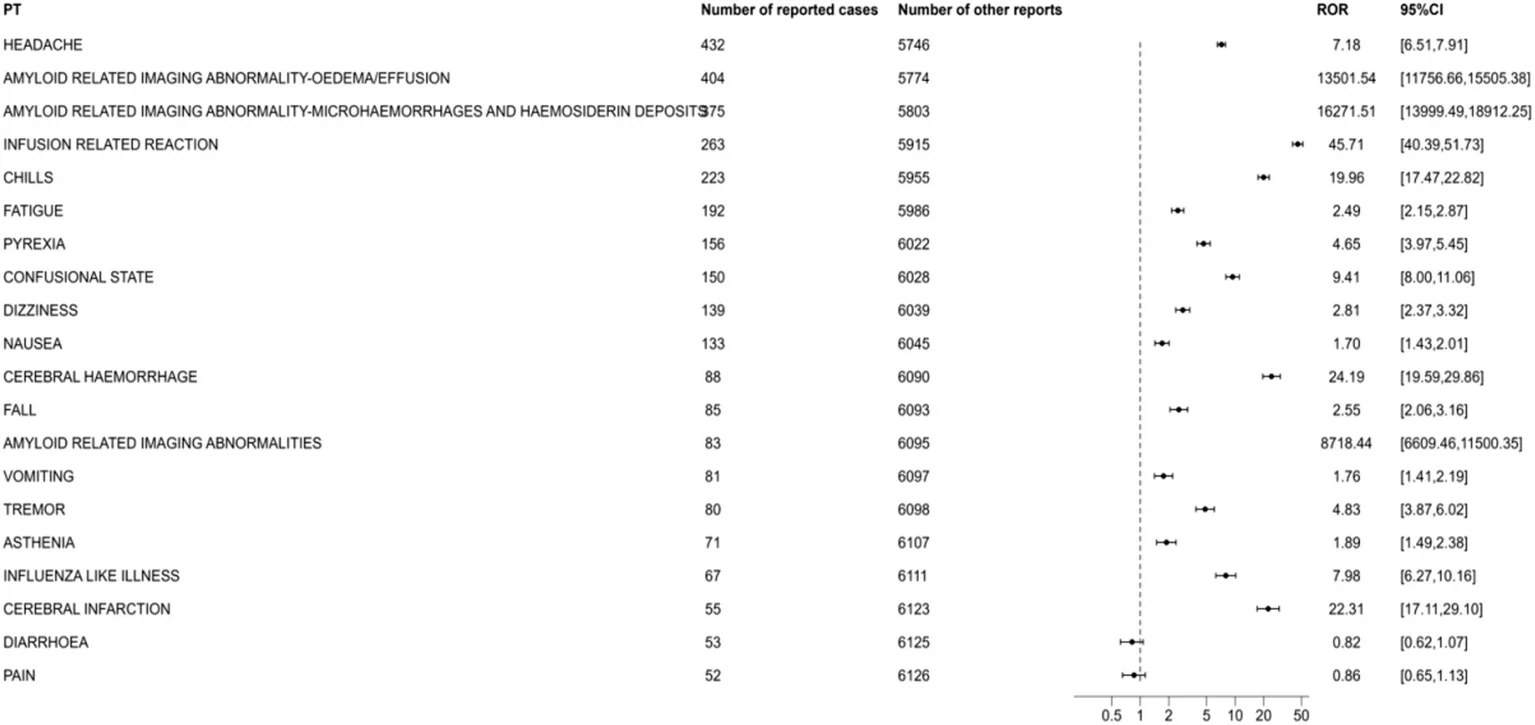
Top 20 adverse events for lecanemab signal in FAERS.

In the single-center real-world prospective observational study, the 13 documented AEs consisted of 7 cases of ARIA-H (12.39 cases/100 patient-years [PYs]), 5 cases of IRRs (8.85/100 PYs), and 1 case of rash (1.77/100 PYs). According to the lecanemab-specific severity scale, the 7 ARIA-H cases were graded as 5 mild, 2 moderate, and none severe. These principal AEs observed in the real-world cohort were qualitatively aligned with the signals identified from the FAERS database as a reference. Moreover, the adverse drug reactions delineated in the official prescribing information for lecanemab were broadly consistent with the top 20 AEs detected in our FAERS-based signal analysis.

Notably, cerebral infarction, a severe cerebrovascular event, is not documented in the official prescribing information. Nevertheless, this specific adverse reaction exhibited a robust signal strength (ROR = 22.31, 95% CI: 17.11–29.10) with 55 reported cases. To further contextualize this signal, we performed a subgroup analysis mapping the concomitant medications and medical histories of these 55 cases. Our data mining revealed that 17 patients (30.9%) had at least one identifiable traditional stroke risk factor or were on concomitant antithrombotic therapy. Specifically, 6 patients (10.9%) were concurrently using antithrombotic agents (e.g., aspirin, clopidogrel, or prasugrel). Furthermore, hypertension was identified in 11 patients (20.0%) and diabetes mellitus in 2 patients (3.6%). Notably, 50 out of the 55 cases (90.9%) reported “product used for unknown indication” for their concomitant medications, underscoring the severe underreporting of baseline comorbidities inherent to the FAERS database. Therefore, while this indicates a potential unlabeled safety signal, the observed cerebral infarction events are likely heavily confounded by preexisting cardiovascular vulnerabilities and concomitant antithrombotic use, necessitating careful clinical interpretation. Concurrently, within the real-world prospective observational cohort, two patients experienced hypotension (co-occurring with an infusion-related reaction) and rash, respectively. Although the signal strengths of these two AEs did not rank within the top 20, they equally merit vigilant monitoring by clinicians.

### 3.3 Analysis of the top three adverse events within the top 10 SOCs by signal frequency for lecanemab

We analyzed the AE reports identifying lecanemab as the primary suspect drug from the FAERS spontaneous reporting database and mapped the corresponding Preferred Terms (PTs) to their respective System Organ Classes (SOCs). To enhance visual interpretation, we generated a forest plot depicting the top 10 SOCs with the highest report frequencies, alongside an analysis of the top three AEs within each SOC (Figure 2). In terms of system organ distribution, AEs associated with lecanemab as the primary suspect drug encompassed 26 SOCs. The most prominently associated category was Nervous System Disorders (ROR = 7.12, 95% CI: 6.77–7.49; PRR = 4.70), which correspondingly accounted for the highest number of reports (*n* = 2,441). This was followed by General Disorders and Administration Site Conditions (ROR = 1.03, 95% CI: 0.96–1.10; PRR = 1.02) and Injury, Poisoning and Procedural Complications (ROR = 0.71, 95% CI: 0.65–0.78; PRR = 0.73); however, neither of these latter two SOCs yielded a positive safety signal based on the FAERS data.

**Figure 2:**
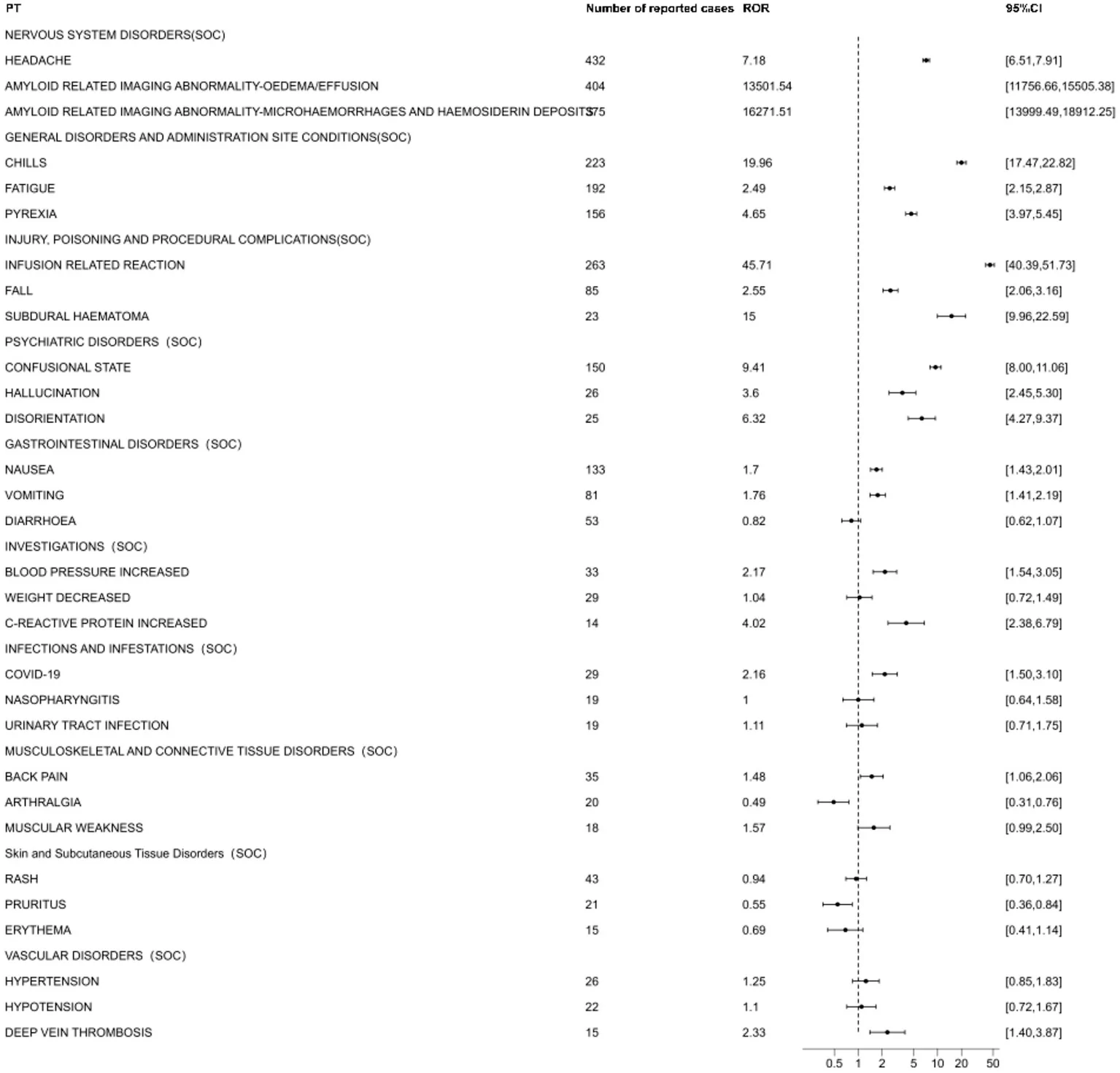
Analysis of the Top 3 Adverse Drug Reactions under the Top 10 SOCs for lecanemab.

Within the Nervous System Disorders SOC, the most frequently reported events were headache (ROR = 7.18, 95% CI: 6.51–7.91), ARIA-E (ROR = 13501.54, 95% CI: 11756.66–15505.38), and ARIA-H (ROR = 16271.51, 95% CI: 13999.49–18912.25). Headache represented the most frequently reported AE in this category, with 432 cases. Under Psychiatric Disorders, the reported AEs included confusional state, hallucination, and disorientation. Notably, confusional state exhibited the strongest signal (ROR = 9.41, 95% CI: 8.00–11.06), comprising 150 reported cases. Other events such as insomnia and agitation were also reported but failed to meet the threshold for a positive signal.

Among the top 10 SOCs ranked by report volume in the FAERS database, Infections and Infestations accounted for a substantial proportion (ranking 7th). Nevertheless, it presented a protective signal (ROR = 0.49, 95% CI: 0.42–0.57), suggesting that lecanemab does not significantly elevate the risk of infection. Similarly, Gastrointestinal Disorders ranked high in report frequency (5th); however, it also yielded a protective signal (ROR = 0.65, 95% CI: 0.59–0.73). This finding diverges from the expected common adverse reactions (e.g., nausea, diarrhea) delineated in the prescribing information, a discrepancy potentially attributable to reporting bias inherent to spontaneous reporting systems.

In summary, based on FAERS data, Nervous System Disorders emerged as the most prominent SOC associated with lecanemab, with a reporting volume vastly exceeding that of other systems. This was predominantly driven by headache, ARIA-related events (edema and microhemorrhages), dizziness, and confusional state. Within General Disorders and Administration Site Conditions, the report volume for “infusion-related reactions” was remarkably high, demonstrating a robust signal (ROR = 45.71, 95% CI: 40.39–51.73) with 263 cases, which aligns with the established risk of infusion reactions. Although Psychiatric Disorders and Gastrointestinal Disorders accumulated a considerable number of reports, their signal strengths remained weak (with RORs approximating or slightly exceeding 1), indicating that these events might be confounded by underlying comorbidities or other contributing factors. While the report frequency for infection-related SOCs (such as Infections and Infestations) ranked among the highest, no statistically positive signal was detected (ROR = 0.49), further corroborating that lecanemab does not significantly augment infection risk.

### 3.4 Time-to-onset (TTO) analysis of lecanemab-related adverse reactions

Based on the time-to-onset (TTO) data for lecanemab-associated adverse events (AEs) extracted from the FAERS database, and following the exclusion of aberrant or spurious reports, a total of 898 AE reports contained valid onset time information, representing approximately 30.2% of all lecanemab-related reports in the database. To assess the representativeness of this subset, we compared its baseline characteristics with the full FAERS dataset. When excluding missing values, the demographic distribution of the TTO subset was highly consistent with the overall dataset: females accounted for 60.2% in the subset versus 59.0% in the full dataset, and the 65–85 age group represented 85.1% in the subset versus 85.7% in the full dataset. The top adverse events in the subset (ARIA-E, ARIA-H, headache, and infusion-related reactions) also perfectly mirrored those of the full dataset. However, ARIA-related events were slightly enriched in the valid subset, likely because asymptomatic imaging findings are typically documented with precise MRI scan dates by healthcare professionals. Therefore, while this subset serves as a demographically representative proxy for temporal trends, it is inherently subject to selection bias, as medically attended events are more likely to have precise dates recorded. The median TTO was 40 days (interquartile range [IQR], 14–118 days). As illustrated in Figure 3, the onset of lecanemab-related AEs is distributed across a broad temporal spectrum, with some cases emerging more than a year post-initiation. However, the vast majority of cases manifested within the first 1 to 2 months following the initiation of lecanemab treatment (*n* = 393, 45.65%). The number of cases occurring in the 3rd month (*n* = 91, 10.57%) and between the 4th and 6th months (*n* = 113, 13.12%) were comparable. This suggests a high probability of AE occurrence within the first year, necessitating heightened clinical vigilance particularly during the initial 1 to 2 months. In our real-world prospective observational cohort, a qualitatively similar temporal pattern was observed, with the highest proportion of AEs also occurring within the first 2 months after treatment initiation (*n* = 3, 23%). Conversely, the proportion of AEs occurring after more than 1 year of lecanemab treatment decreased significantly in the FAERS data (*n* = 118, 13.70%), a trend that was also noted in our cohort. Collectively, these findings provide supportive real-world evidence for the medium-term safety profile of lecanemab from both the FAERS signal detection and our prospective cohort; however, this evidence should be interpreted with caution given the modest sample size of the cohort and the median exposure duration of 185 days, which limit the ability to draw robust conclusions about long-term safety.

**Figure 3:**
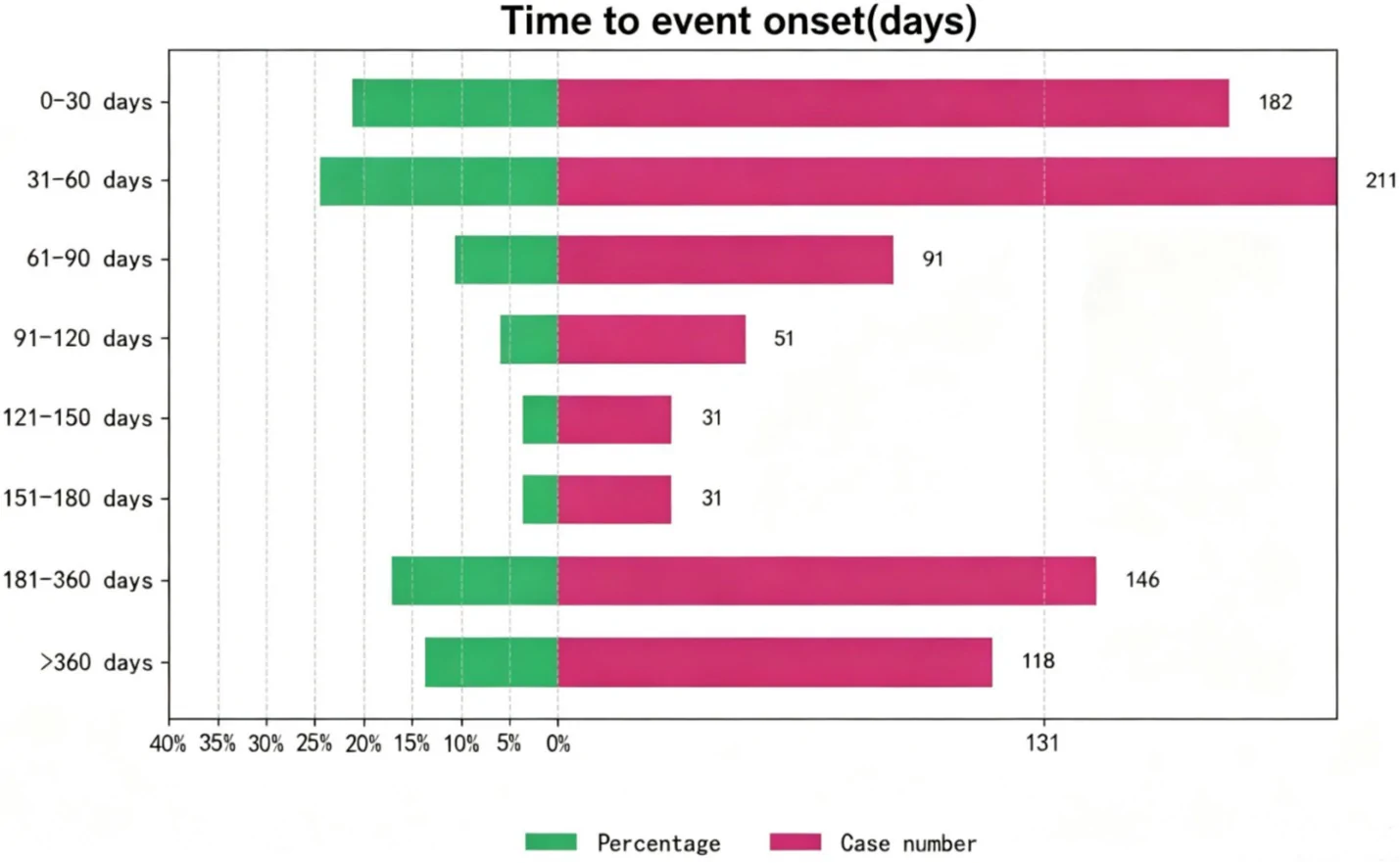
Time-to-onset analysis of adverse events related to lecanemab.

Through the Weibull shape parameter (WSP) test analysis of the TTO profile—computed using the dates of event onset and drug initiation—the AE TTO curve for lecanemab was characterized based on its shape and scale parameters (Table 4). As demonstrated, the shape parameter (α) for lecanemab was < 1, with its 95% CI also exclusively < 1. This classifies the TTO curve of lecanemab-related AEs as an “early failure” profile. In clinical terms, this signifies that the hazard risk of encountering an AE is highest during the early phase of treatment and progressively declines thereafter, notwithstanding a persistent fraction of delayed-onset events (approximately 13.7% occurring after 1 year in the FAERS analysis). Given the limitation of missing TTO data, these Weibull parameter estimates should also be considered exploratory and warrant confirmation in future studies with more complete data.

**Table 4:** Time-to-onset analysis of adverse event signals for lecanemab.

Cases (*n*)	TTO (days)		Scale parameter				Failure type
	Median(IQR)	Min-max	α	95%CI	β	95%CI	
898	49	1–983	88.6023	80.0905–97.3327	0.6792	0.6493–0.7166	Early failure

*n*, number of cases with available time-to-onset; IQR, interquartile range; TTO, Time-to-onset. When TTO is 0 days, the adverse event occurred within the same day with the therapy.

## 4 Discussion

### 4.1 Demographic characteristics of lecanemab-related ADR cases

A total of 2,969 lecanemab-related adverse drug reaction (ADR) reports have been extracted from the FAERS database since 2023. Concurrently, the single-center prospective observational study enrolled 91 patients receiving lecanemab, among whom 13 experienced adverse events. In the FAERS data, the analysis of these AEs revealed a higher proportion of reports in females compared to males, which is consistent with previous studies indicating a higher incidence of AD in females (Li Z. et al., 2025). Regarding geographic distribution, FAERS data showed that North America emerged as the primary reporting region for AEs, accounting for 81.8% of reports, correlating with the region where lecanemab initially obtained regulatory approval. In terms of age distribution, the FAERS data indicated that patients with dementia aged 65 to 85 years constituted the highest proportion of ADR reports (1,918 cases, accounting for 64.6% of FAERS reports). In our cohort, the same age group also accounted for the largest share (10 cases, 76.9%). Both distributions align with the known epidemiological features of this age group, as this age bracket accounts for a substantial proportion of the dementia population and possesses a high number of diagnosed cases (Dhana et al., 2023). A study by Zeng et al. based on the FAERS database investigating donepezil combined with memantine similarly found that 55.1% of patients with dementia reporting AEs fell within the 65–85 age group (Zeng et al., 2025). Therefore, the FAERS data suggest that patients with dementia aged 65 to 85 years represent a predominant demographic receiving this medication and a highly vulnerable group for ADRs. Clinicians should prioritize this population and routinely monitor relevant safety parameters —a recommendation that is also supported by the observations from our cohort.

### 4.2 Risk signal analysis of lecanemab-related ADRs

The screening of lecanemab-related ADR risk signals identified a total of 252 Preferred Terms (PTs) spanning 12 System Organ Classes (SOCs). Except for confusional state, cerebral infarction, and somnolence, the top 20 PTs are explicitly documented in the official prescribing information. Notably, the 13 adverse reactions documented in our single-center prospective cohort were qualitatively encompassed within the top 20 PTs identified from the FAERS data, showing directional consistency rather than serving as numerical validation of the cohort findings. Furthermore, in the FAERS database, the Phase III Clarity AD study reported cerebral hemorrhage in 5 patients, resulting in 3 fatalities (van Dyck et al., 2023; van Dyck et al., 2025). In our FAERS analysis, a total of 119 deaths (4% of FAERS reports) were identified. This suggests that although no fatal events were observed in our single-center prospective study—likely constrained by the limited sample size—the FAERS signals highlight that clinicians must remain highly vigilant regarding lecanemab-induced fatal events, particularly in patients concurrently receiving anticoagulant therapy.

ARIA remains the most scrutinized AE associated with anti-Aβ antibodies. In the CLARITY AD trial, the incidence of ARIA-E was 12.6% and ARIA-H was 17.3% over the 18-month core treatment period (van Dyck et al., 2023). In our single-center prospective cohort, the incidence rates were 0% for ARIA-E and 7.7% for ARIA-H, which are numerically lower than those reported in the pivotal trial. This discrepancy warrants a systematic interpretation considering three potential contributors. First, genotype selection played a critical role—we performed pretreatment APOE genotyping and excluded all ε4 homozygotes carriers, thereby eliminating the subgroup at the highest risk for ARIA-E. Second, the follow-up duration in our real-world cohort was limited, with a maximum of 17 months, whereas the CLARITY AD core study had a prespecified 18-month follow-up (van Dyck et al., 2023). This shorter observation window may have missed late-onset ARIA-E cases. Third, differences in MRI surveillance strategies likely contributed to the observed discrepancy. In CLARITY AD, protocol-mandated safety MRIs were performed at weeks 9, 13, and months 6, 12, and 18, enabling systematic detection of asymptomatic ARIA-E (van Dyck et al., 2023). By contrast, in our real-world practice, while MRI was recommended on 8 occasions over the 36-infusion treatment course, only 4 of these were mandatory; the remaining scans were performed at the clinician’s discretion or when patients developed symptoms suggestive of ARIA. This less intensive surveillance approach may have led to under-detection of asymptomatic or mild ARIA-E.

Importantly, all ARIA events observed in our cohort were mild to moderate and stabilized following conservative management, which we defined as temporary drug suspension (withholding one or two infusions) followed by clinical and imaging re-evaluation; no patient required permanent discontinuation. While previous studies indicate that APOE ε4 carriers face a higher risk of ARIA (Cummings et al., 2023), our study addressed this by conducting pretreatment genotyping and excluding all APOE ε4/ε4 homozygotes, while retaining APOE ε4 heterozygotes in the cohort. This genotyping-based selection strategy resulted in a lower baseline ARIA risk for our cohort. Accordingly, a 0% incidence of ARIA-E does not indicate an absence of risk in this population; rather, it suggests a relatively lower short-term risk. Continuous imaging monitoring remains necessary during treatment, and our findings should be interpreted as specific to this selected population and are not generalizable to the entire Alzheimer’s disease patient population.

Regarding Nervous System Disorders, aside from the common AEs of headache and ARIA, FAERS analysis identified cerebral infarction—a severe cerebrovascular event—which is not documented in the prescribing information, highlighting a potentially critical unlabeled safety signal. Mechanistically, lecanemab, as an anti-Aβ monoclonal antibody, functions by binding to and clearing Aβ plaques in the brain (Cummings et al., 2016; Gibson et al., 2024). However, Aβ deposition on blood vessel walls (cerebral amyloid angiopathy, CAA) is prevalent among AD patients. Anti-Aβ therapy may rapidly clear vascular Aβ, leading to the disruption of vascular integrity, triggering inflammatory responses, and increasing vascular permeability, which may subsequently induce thrombosis and result in cerebral infarction (Gibson et al., 2024; Alammar et al., 2025). The FAERS analysis demonstrated that while the incidence of cerebral infarction is low in the lecanemab-treated population (55 cases in this dataset, representing 0.89% of total ADR reports), the signal strength is extremely robust (ROR > 22). However, as our subgroup analysis revealed, nearly one-third of these patients had identifiable traditional stroke risk factors (e.g., hypertension, diabetes) or were using antithrombotic agents, despite the severe underreporting of medical history in FAERS. It is highly plausible that underlying severe CAA, combined with concomitant anticoagulant/antiplatelet therapy and preexisting cardiovascular comorbidities, significantly contributed to these ischemic events. Therefore, while cerebral infarction emerged as a potential safety signal associated with lecanemab, it requires careful contextualization. However, it is important to emphasize that the signal detected in the FAERS analysis does not establish a causal relationship with lecanemab treatment and should be interpreted as a hypothesis-generating finding that warrants further pharmacovigilance, prospective monitoring, and mechanistic studies to confirm this association.

Additionally, within Psychiatric Disorders, confusional state exhibited the strongest signal, aligning with the prescribing information. Some patients also presented with symptoms such as hallucinations and disorientation, which may be intertwined with the natural progression of AD. Several previous studies have established that as the disease progresses, AD patients may experience hallucinations, sleep disturbances, and disorientation (Gibson et al., 2025; Chen et al., 2024; Laczó et al., 2025). Nevertheless, a potential causal association between lecanemab and these psychiatric symptoms cannot be entirely ruled out and warrants continuous monitoring.

Headache and infusion-related reactions (IRRs) were also common in the real-world cohort, consistent with the prescribing information. The majority of IRRs manifested as transient pyrexia, chills, or blood pressure fluctuations. This is likely because lecanemab, functioning as a large-molecule exogenous protein, can directly trigger a cascade of immune system responses, manifesting as pyrexia and chills (Paczynski et al., 2025; Asmani et al., 2024; Xing et al., 2025). Research indicates that implementing management strategies can effectively reduce the incidence of IRRs (Paczynski et al., 2025). In clinical practice, preemptive administration of loratadine and acetaminophen/paracetamol prior to infusion has been shown to significantly decrease the incidence of IRRs from 37 to 27% (Paczynski et al., 2025).

Regarding IRRs, the single-center study reported 1 case of a post-infusion hypotensive event (1.77/100 PYs). FAERS analysis revealed an ROR for transient hypotension of 1.10 (95% CI: 0.72–1.67), which failed to meet the threshold for a positive signal, indicating that lecanemab does not significantly elevate the risk of hypotension based on FAERS data. The hypotensive event observed in the single-center study was more likely related to the patient’s suboptimal baseline blood pressure control.

### 4.3 Time-to-onset characteristics of lecanemab-related ADRs and conclusions

The statistical analysis of the time-to-onset (TTO) for ADRs following lecanemab administration revealed a high probability of AEs occurring within the first year, necessitating particular clinical vigilance during the first 1 to 2 months. IRRs (such as headache, chills, pyrexia, nausea, and rash) typically occurred during the infusions of the first two treatment sessions (with exposure times generally ranging from 1 to 14 days). Conversely, ARIA (including ARIA-E and ARIA-H) occurred across all phases of treatment, suggesting no definitive correlation between its onset and treatment duration. Symptoms such as headache and confusional state frequently emerged within 14 days post-administration, prompting clinicians to conduct rigorous monitoring for these symptoms during the early phase of treatment. Meanwhile, ARIA-related AEs necessitate continuous neuroimaging monitoring throughout the entire treatment cycle, which aligns with findings from prior research (Cummings et al., 2023).

By performing signal mining and analysis of lecanemab-related AE reports from the FAERS database and cross-validating the findings with a single-center real-world prospective observational study, the primary findings are as follows: (1) The types of AEs observed in our real-world cohort were qualitatively consistent with those reported in the Phase III Clarity AD study, with ARIA-E, ARIA-H, headache, and IRRs being the most prominent events; (2) FAERS analysis revealed that cerebral infarction, a severe cerebrovascular event, is an unlabeled potential safety signal that merits heightened clinical attention; (3) FAERS analysis identified that patients with dementia aged 65 to 85 years represent a highly susceptible demographic for AEs, demanding special vigilance during the first 2 months of treatment. Notably, ARIA (including both ARIA-E and ARIA-H) can occur at any stage throughout the therapeutic course.

The limitations of this study include its single-center design and limited sample size. Additionally, the FAERS analysis is inherently subject to spontaneous reporting bias, precluding the calculation of true incidence rates. Furthermore, the TTO analysis was based on only 861 reports with valid onset times, representing approximately 29% of all lecanemab-related FAERS reports; this subset may not be fully representative of the entire dataset, and missing onset-time records may introduce selection bias. Accordingly, the TTO findings should be interpreted as preliminary signal observations and warrant confirmation in future studies with more complete data. Furthermore, the median lecanemab exposure duration in our prospective cohort was 185 days, which primarily informs the medium-term safety profile rather than long-term safety beyond 12 months. While the open-label extension of the Clarity AD trial has provided evidence of lecanemab’s long-term safety and tolerability over 36 months (van Dyck et al., 2023), our real-world data contribute complementary evidence on the earlier treatment phase, particularly regarding ARIA monitoring and management in routine clinical practice. Nevertheless, the relatively short observation period in our cohort precludes robust conclusions about rare or delayed adverse events that may emerge with prolonged exposure, such as long-term ARIA progression or other cumulative toxicities. Future large-scale prospective multicenter studies are imperative to validate these findings and confirm the identified potential safety signals, thereby mitigating clinical medication risks and effectively reducing the incidence of adverse events.

## 5 Conclusion

In conclusion, by integrating findings from the FAERS signal detection with observations from our real-world prospective cohort, this study suggests that lecanemab exhibits a generally manageable safety profile, with the AE types observed in our cohort being broadly aligned with those reported in pivotal clinical trials. ARIA and infusion-related reactions constitute the most prevalent adverse events. FAERS analysis further indicated that patients aged 65 to 85 years represent a highly susceptible demographic, necessitating rigorous clinical monitoring, particularly during the initial two months of therapy, while ARIA requires continuous surveillance throughout the entire treatment course. Crucially, our FAERS-based analysis identified cerebral infarction as a robust, unlabeled potential safety signal. While this underscores the need for vigilance regarding cerebrovascular complications, clinicians must carefully consider the confounding roles of concomitant antithrombotic use and traditional stroke risk factors in this elderly demographic. However, given the relatively short median exposure duration of 185 days in our prospective cohort, the long-term safety profile of lecanemab beyond 12 months remains to be fully elucidated. Moving forward, large-scale, multicenter prospective studies are imperative to validate these emerging safety signals and to optimize risk-mitigation strategies for lecanemab in real-world settings.

## Data Availability

The datasets presented in this study can be found in online repositories. The names of the repository/repositories and accession number(s) can be found at: https://www.fda.gov/drugs/fdas-adverse-event-reporting-system-faers/fda-adverse-event-reporting-system-faers-public-dashboard.
